# Homoplantaginin inhibits the progression of ulcerative colitis in mice by regulating the MMP9-RLN2 signaling axis

**DOI:** 10.3389/fmed.2025.1582066

**Published:** 2025-06-02

**Authors:** Yu Tao, Rongrong Shao, Mengting Cui, Haojie Wang, Manman Xiang, Sitang Ge, Min Deng, Xian Li, Fang Liu, Fangtian Fan

**Affiliations:** ^1^School of Pharmacy, Bengbu Medical University, Bengbu, China; ^2^Department of Electrocardiology, First Affiliated Hospital of Bengbu Medical University, Bengbu, China; ^3^Digestive Tract Disease, First Affiliated Hospital of Bengbu Medical University, Bengbu, China; ^4^Anhui Engineering Technology Research Center of Biochemical Pharmaceutical, Bengbu, China

**Keywords:** homoplantaginin, ulcerative colitis, MMP9, RLN2, inflammatory response

## Abstract

**Introduction:**

Ulcerative colitis (UC) is a chronic inflammatory bowel disease characterized by colonic mucosal inflammation and ulceration. This study investigates the therapeutic effects of homoplantaginin (Homo), a flavonoid derived from *Salvia plebeia* R. Brown, on dextran sulfate sodium (DSS)-induced colitis in mice, as well as its underlying mechanisms of action.

**Methods:**

In this study, a mouse colitis model was established using DSS to assess the remission effect of Homo on colitis mice. Quantitative reverse transcription PCR (qRT-PCR) was employed to investigate the impact of Homo on intestinal mucosal barrier and pro-inflammatory cytokines in mice. The possible target genes of Homo were analyzed and screened using bioinformatics and molecular docking approaches. Microscale Thermophoresis (MST) technique was employed to examine the binding interaction between Homo and its target gene, matrix metalloproteinase 9 (MMP9). Finally, the combination of Homo and MMP9 inhibitors was utilized to verify whether Homo alleviates DSS-mediated colitis in mice through modulation of MMP9.

**Results:**

Homo (50 mg/kg) significantly alleviated colitis symptoms, lowered myeloperoxidase (MPO) activity, and improved histopathological outcomes. qRT-PCR analysis revealed that Homo inhibited the expression of pro-inflammatory cytokines (TNF-*α*, IL-1β, IL-6 and IFN-*γ*) and related molecules, highlighting its anti-inflammatory properties. Additionally, Homo strengthened the intestinal mucosal barrier by regulating barrier protein expression. Bioinformatics analysis identified that MMP9 as a potential target of Homo, while molecular docking and MST analysis revealed a dose-dependent inhibition of MMP9. Moreover, the MMP9/Relaxin 2 (RLN2) signaling pathway was implicated in Homo’s effects, as evidenced by the upregulation of RLN2 mRNA upregulation and its interaction with MMP9. The combination of the MMP9 inhibitor IN-1 with Homo demonstrated no synergistic effect, it confirmed the role of the MMP9-RLN2 axis in colitis modulation.

**Conclusion:**

Homo demonstrates significant potential in alleviating colitis through targeting the MMP9-RLN2 signaling pathway, warranting further clinical investigation in UC treatment.

## 1 Introduction

Ulcerative colitis (UC) is a common chronic inflammatory bowel disease (IBD) characterized by progressive inflammation and injury of the colon and rectal mucosa. However, its exact etiology of UC has not been fully elucidated. The main feature is progressive inflammation and injury of the colon and rectal mucosa. UC can lead to abdominal pain, diarrhea, hematochezia and other clinical symptoms, significantly affecting the patients’ quality of life and may increase the risk of colon cancer (1). At present, UC treatment includes 5-aminosalicylate (5-ASA), glucocorticoids and immunosuppressive agents. However, these therapies exhibit limited efficacy in certain patients and may be associated with severe side effects (2). Therefore, finding novel therapeutic targets and drugs has become a critical area of UC research.

Matrix metalloproteinase 9 (MMP-9) plays a key role in inflammation and tissue remodeling, and its up-regulation is considered to be closely related to the occurrence and development of a variety of inflammatory diseases (3). MMP-9 can degrade the extracellular matrix and promote cell migration, playing an important role in maintaining the inflammatory microenvironment and promoting the infiltration of inflammatory cells (4–6). Studies have demonstrated the high expression of MMP-9 is closely associated with the degree of inflammation in patients with UC. Inhibition of MMP-9 activity may contribute to reducing the damage and inflammatory response of colitis (7, 8). In addition, Relaxin (RLN), a bioactive peptide, has been demonstrated to have a protective effect in pathological conditions such as colitis. Studies have revealed its potential to improve the remodeling process of colonial tissue by regulating extracellular matrix components as well as inhibiting the MMP expression (9, 10). Consequently, the interaction between MMP-9 and RLN may play a crucial role in regulating inflammatory response and could provide novel ideas for UC treatment.

Homoplantaginin (Homo), a natural bioactive compound derived from the Chinese medicine *Salvia plebeia* R. Brown, has recently garnered increasing attention. Studies have demonstrated that Homo exhibits various pharmacological properties, including anti-inflammatory and anti-oxidant effects (11, 12). However, the effects of Homo on UC have not yet been reported. In addition, the specific relationship between Homo, MMP-9, and RLN signaling pathways in UC remains unexplored. As a result, this study aimed to investigate the mechanism of Homo in DSS-induced UC in mice, and to analyze its effects on MMP-9 and RLN signaling pathways to provide both a theoretical basis and experimental foundation for UC treatment.

## 2 Materials and methods

### 2.1 Chemical reagents

Homo (C22H22O11, MW: 462.4, HPLC ≥ 98%) was procured from Shanghai Yuanye Bio-Technology Co., Ltd. (Shanghai, China). Dextran sulfate sodium (DSS, molecular weight 36–50 kDa) was procured from MP Biomedical (Aurora, OH, USA). The myeloperoxidase (MPO) activity assay kit was obtained from Nanjing JianCheng Bioengineering Institute (Nanjing, China). MMP-9 proteins and antibodies against MMP9 were procured from Abcam (Cambridge, UK). MMP-9-IN-1(IN-1) was acquired from Shanghai Haoyuan Biomedical Technology Co., Ltd. (Shanghai, China).

### 2.2 Experimental animals and study design

Female C57BL/6J mice (18–22 g), 6–8 weeks old, were procured from the Comparative Medicine Center of Yangzhou University (Yangzhou, China). They were housed under a 12 h light/dark cycle (21 ± 2°C), and provided with a standard chow diet and water *ad libitum*. The animal experiments were approved by the Ethics Committee of Bengbu Medical University (Approval No. 2024–525), and all animals received humane care in accordance with the National Institutes of Health Guidelines.

Dextran sulfate sodium-induced colitis was induced as previously described (13). Female C57BL/6J mice were fed with 2.5% DSS (dissolved in sterile distilled water) for 7 days, followed by sterile distilled water alone for an additional 3 days. The mice were randomly assigned to the following groups: Normal group, DSS group, Homo (12.5, 25, and 50 mg/kg) group, and 5-ASA (200 mg/kg) group. Homo and 5-ASA were orally administered from day 1 to day 10. On day 10, mice were sacrificed, and their colons were collected and photographed.

### 2.3 Disease activity index (DAI)

The disease activity index (DAI) was calculated as previously described (14). Briefly, the scoring criteria were as follows: (a) body weight loss: 0 = none; 1 = 1–5%; 2 = 5–10%; 3 = 10–15%; 4 = over 15%; (b) stool consistency: 0 = normal; 2 = loose stools; 4 = diarrhea; (c) gross bleeding: 0 = normal; 2 = hemoccult; 4 = gross bleeding.

### 2.4 MPO activity

The MPO activity in the colons of mice with DSS-induced colitis was measured by using commercial kits following the manufacturer’s instructions.

### 2.5 Histopathological analysis

The distal ends of the colons isolated from mice with DSS-induced colitis were fixed in 10% formalin, embedded in paraffin, sectioned (5 μm), and stained with hematoxylin and eosin (H&E) for histological evaluation. The histological scoring was performed as previously described (13). The histologic changes of colons were assessed by a pathologist blinded to the experimental groups. The total severity score was calculated by summing the individual parameter scores.

### 2.6 Quantitative real-time quantitative PCR (RT-qPCR) assay

Total RNA was extracted from colon tissues using the TRIzol reagent following the manufacturer’s instructions. The specific detection steps of RT-qPCR were carried out as previously described (13). The primer sequences used are listed in Table 1. The expression of each gene was normalized to GAPDH, and calculated by using the 2^−△△^Ct method.

**Table 1:** Oligonucleotide sequences of quantitative real-time PCR.

Gene	Primer	Sequences (5′-3′)
TNF-α	Forward	CCCTCAGCGAGGACAGCAAG
	Reverse	ACAGAACCTGCCTGGTTGGC
IL-1β	Forward	GCTGTGGAGAAGCTGTGGCA
	Reverse	TGGGAACGTCACACACCAGC
INF-γ	Forward Reverse	CTGCTGATGGGAGGAGATGT TGTCATTCGGGTGTAGTCACA
IL-6	Forward Reverse	CGGAGAGGAGACTTCACAGAG ATTTCCACGATTTCCCAGAG
MIP-2	Forward Reverse	GGCAAGGCTAACTGACCTGGAAAGG ACAGCGAGGCACATCAGGTACGA
MCP-1	Forward Reverse	GTGTTGGCTCAGCCAGATGC GACACCTGCTGCTGGTGATCC
E-Selectin	Forward Reverse	TGCATGGCTCAGCTCAACTTGA CACTGTGCCGAAAACTGCTGTTC
VCAM-1	Forward Reverse	ATCTGGGTCAGCCCCTCTCCTATAC TGTCTGCTCCACAGGATTTTGG
ICAM-1	Forward Reverse	TGAATGCCAGCTCGGAGGATCAC CGTGCAGTTCCAGGGTCTGGTT
MAdCAM-1	Forward Reverse	TGTCTGCTCCACAGGATTTTGG CCCTGGCCCTAGTACCCTAC
CLDN-2	Forward Reverse	CTTGGACCGTTGAGGGAAGG CGACTGCCCCCTTAACTCTC
Occludin	Forward Reverse	GTGAATGGGTCACCGAGGG AGATAAGCGAACCTGCCGAG
ZO-1	Forward Reverse	ACTCTTCAAAGGGAAAACCCGA GCAAAAGACCAACCGTCAGG
MUC2	Forward Reverse	CCCGAAGATTCCTGCTGGTT AGAGGAAACAGGAGTGGGGT
BCL2	Forward Reverse	TGGGATGCCTTTGTGGAACT TTGGCAATTCCTGGTTCGGT
PTGS2	Forward Reverse	AGCCCATTGAACCTGGACTG ACCCAATCAGCGTTTCTCGT
AKT1	Forward Reverse	TCGGAGTAGGAGCAGGAAGTG GGTCGTGGGTCTGGAATGAG
CCL2	Forward Reverse	CCTGCTGCTACTCATTCACCA ATTCCTTCTTGGGGTCAGCA
ALB	Forward Reverse	TGCTTTTTCCAGGGGTGTGT CATGGTGTCATGCTCCACCT
MMP9	Forward Reverse	GCCGACTTTTGTGGTCTTCC TGGCCTTTAGTGTCTGGCTG
CASP3	Forward Reverse	GGGGAGCTTGGAACGCTAAG CCGTACCAGAGCGAGATGAC
TP53	Forward Reverse	ATATCAGCCTCGAGCTCCCT CAGGCACAAACACGAACCTC
MAPK3	Forward Reverse	CCAGGCCTAACCCTCTCTCT GATACAGGCACGGGGAGATG
RLN1	Forward Reverse	GAGCCTTTCGATACGACGCT GCTGGCTCATCAATCCACCA
RLN2	Forward Reverse	TTTGGGCCCTTCCTGGAAAT AAGGTGTTGCCTTCAGCTCC
RLN3	Forward Reverse	AGATGTGTTGGCTGGCCTTT CGCTTCTCCATTGCTCAACC
GAPDH	Forward Reverse	GGTGAAGGTCGGTGTGAACG CTCGCTCCTGGAAGATGGTG

### 2.7 Bioinformatics analysis

#### 2.7.1 Component target prediction

The corresponding sdf format files of the active ingredient Homoplantaginin (Homo) were downloaded from the Pub Chem database^1^ and imported into the CTD database^2^ and Swiss database^3^ to search for the corresponding targets of Homo to obtain the target proteins corresponding to the predicted compounds.

#### 2.7.2 Disease target prediction

UC-related targets were searched in the Gene Cards database^4^ and Omim^5^ with ‘ulcerative colitis ‘as the name of the disease. The targets obtained from the two disease databases were merged, and duplicate entries were removed to derive the final set of UC-related targets.

#### 2.7.3 Construction of protein–protein interaction network (PPI)

To elucidate the interaction between the related targets of Homo and those associated with UC, the online mapping platform^6^ was utilized to screen out the intersection targets and generate the Venn diagram. Subsequently, the intersection targets were constructed on the STRING platform^7^ to construct a PPI network. The species was set to ‘*Homo sapiens*’, and the minimum interaction threshold was defined as ‘confidence medium = 0.4’. All other parameters were kept at their default settings to obtain the PPI network. The first 10 disease targets were finally obtained by the MCC algorithm of Cytoscape 3.7.2 plug-in CytoHubba (Cytoscape plug-in).

#### 2.7.4 GO and KEGG pathway enrichment analysis

The mapping results between Homo and UC targets were imported into the online software metascape^8^, with *H. sapiens* was set as the reference species. A significance threshold of *p* < 0.05 was selected. The results were analyzed by GO and KEGG by selecting GO biological processes, GO molecular functions, GO cellular components, and KEGG pathway.

#### 2.7.5 Component-target-pathway network construction

Cytoscape 3.7.2 was utilized to construct the network diagram of the Homo-UC disease target-pathway, and the plug-in ‘Network Analysis’ function of Cytoscape 3.7.2 software was employed to evaluate the network topology parameters of active components and targets, including degree, betweenness and closeness. Based on the network topology parameters, the core targets and the key active components responsible for therapeutic effects were identified.

### 2.8 Molecular simulation assay

The crystal structure of ALB(2n0x), CCL2(4usp), MAPK3(4qtb), BCL2(1g5m), MMP9(6esm), PTGS2(1pxx), TNF(1a8m), AKT1(1unq), TP53(2k8f) and CASP3(1cp3) complexed with estradiol was downloaded from the PDB database, and then processed using the ‘prepare_receptor4.py’ script in ‘AutoDockTools 1.5.6’, whose process mainly involved the removal of crystal water, ions, and non-standard amino acid residues. The three-dimensional structure of Homo was handled using the ‘prepare_ligand4. py’ script in ‘AutoDock Tools 1.5.6’, which primarily involved the removal of non-polar hydrogen and the giving of the atomic type and Gasteiger charge. Next, the above 10 crystal structures and Homo were analyzed through the ‘AutoDock Vina 1.1.2’ program. The binding free energy, action of hydrogen bonds, and hydrophobic and electrostatic interactions were analyzed. The top 10 ranked conformations of Homo were selected from the output tab by specifying the output numbers.

### 2.9 Microscale thermophoresis (MST)

The KD value was determined using the Monolith NT.115 instrument (NanoTemper Technologies). A range of concentrations of Homo was incubated with 200 nM of purified MMP9 protein for 40 min in assay buffer (50 mM Hepes, 10 mM MgCl_2_, 100 mM NaCl, pH 7.5, and 0.05% Tween 20). The samples were loaded into the NanoTemper glass capillaries, and MST was performed using 100% LED power and 80% MST power. The KD value was calculated using the mass action equation via the NanoTemper software based on duplicate reads of an experiment.

### 2.10 Tissue immunofluorescence staining

Thin tissue sections (5 μm) were blocked with 1% bovine serum albumin and subsequently incubated with primary antibodies overnight at 4°C. Following three washes with phosphate-buffered saline, the sections on slides were incubated with fluorescence-conjugated secondary antibodies at room temperature for 2 h. The sections incubated with secondary antibodies alone served as a negative control. Subsequently, the sections were stained with 4′, 6-diamidino-2-phenylindole (DAPI, Bioword, China) at a concentration of 0.1 μg/mL for 10 min. Finally, the images were captured using an Olympus IX51.

### 2.11 Statistical analysis

Data are presented as means ± SEM. Student’s t-test was used to compare the mean differences between the two groups, while a one-way ANOVA followed by the LSD test was employed to compare the mean differences between multiple groups. A *p*-value < 0.05 was considered statistically significant.

## 3 Results

### 3.1 Homo alleviates DSS-induced colitis in mice

**Figure 1:**
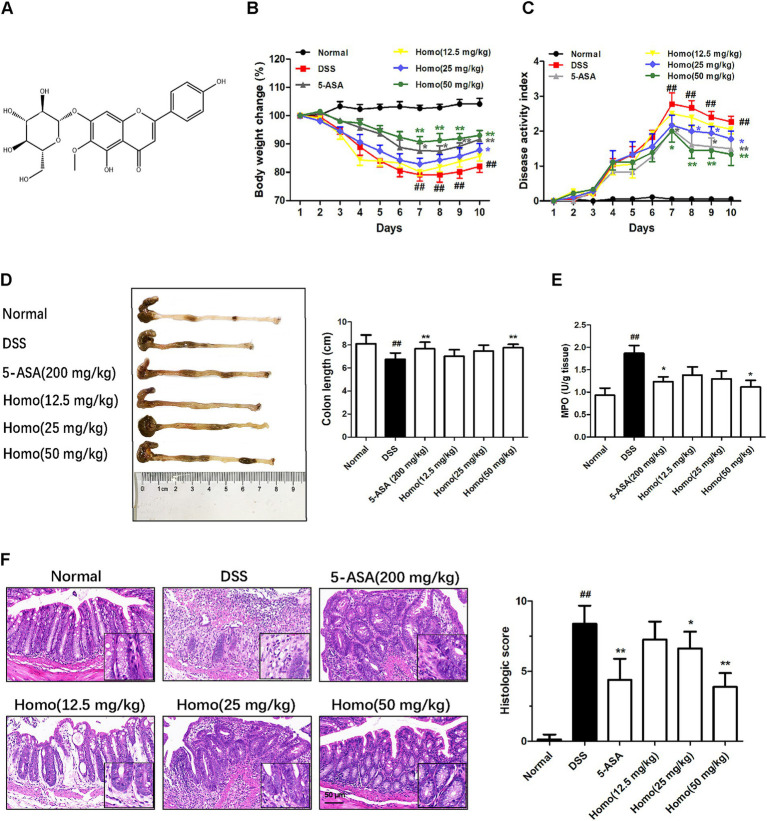
Homo attenuated DSS-induced colitis in mice. **(A)** The chemical structure of Homo. **(B)** Percent change of body weight. **(C)** DAI scores. **(D)** The length of colons. **(E)** MPO activity in colon tissues. **(F)** H&E staining and histological score of colon tissues (scale bar, 50 μm). (*n* = 6–8). *^##^p* < 0.01 vs. Normal group; *p < 0.05 and **p < 0.01 vs. DSS group. Homo, homoplantaginin.

The chemical structure of Homo is illustrated in Figure 1A. A DSS-induced mouse colitis model was established to investigate the effects of Homo on the colon of mice. Mice in the DSS group experienced varying degrees of body weight loss, loose stools, and fecal occult blood in comparison to the normal control group. Homo (50 mg/kg) and 5-ASA (200 mg/kg) demonstrated good therapeutic effects in alleviating these symptoms (Figures 1B,C). Moreover, Homo (50 mg/kg) and 5-ASA (200 mg/kg) significantly inhibited colon shortening (Figure 1D) and reduced MPO activity in colon tissue (Figure 1E). Pathological examination revealed that, compared to the normal group, the model group exhibited significant epithelial damage and inflammatory cell infiltration (Figure 1F). Treatment with Homo (50 mg/kg) and 5-ASA (200 mg/kg) demonstrated notable improvement in these pathological changes. These findings suggest that Homo can significantly alleviate the progression of colitis in mice.

### 3.2 Homo inhibits the expression of pro-inflammatory factors in the colon of colitis mice

**Figure 2:**
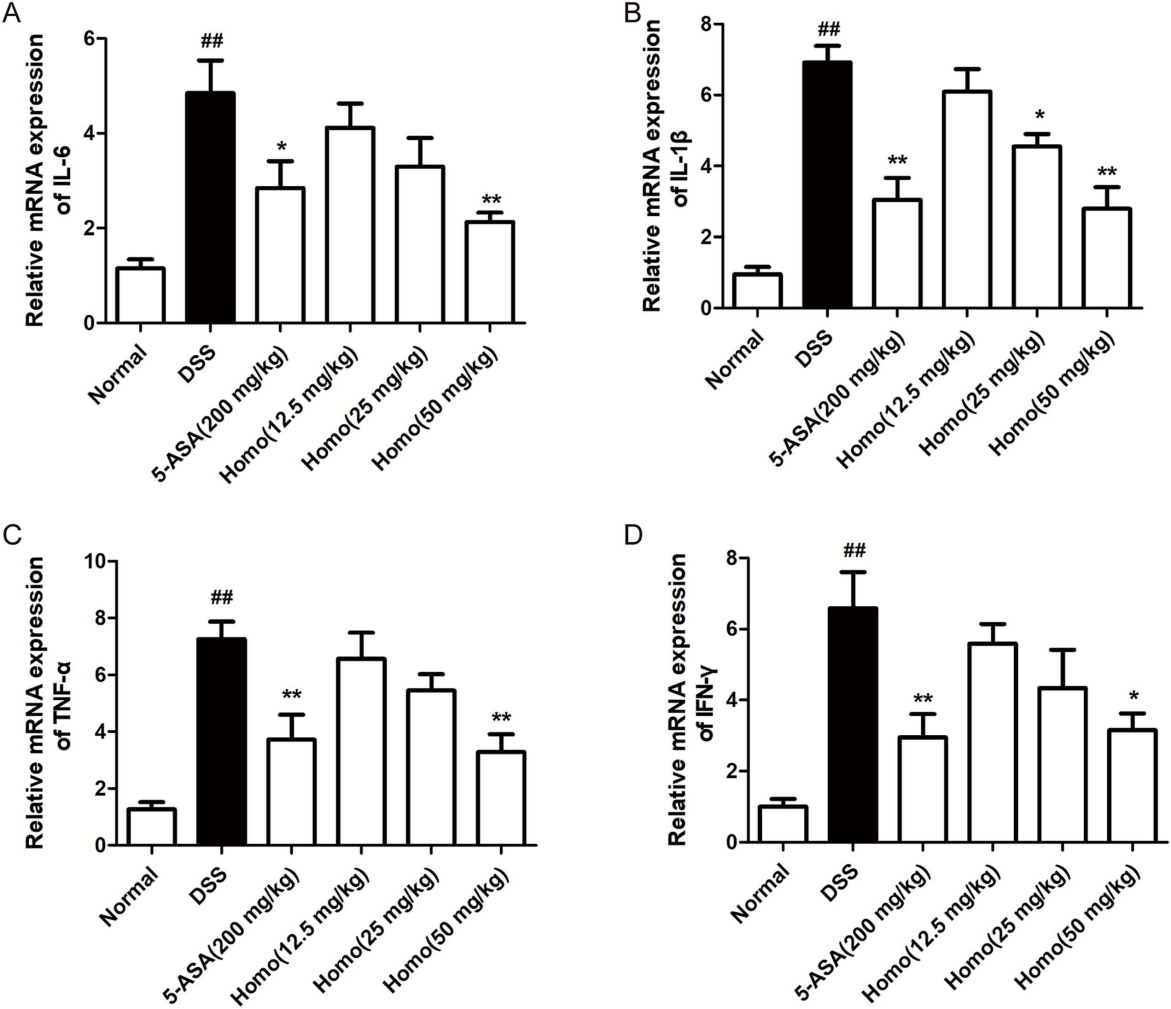
Homo attenuated the expression of pro-inflammatory factors in colitis tissues of mice. The mRNA leves of **(A)**TNF-*α*, **(B)** IL-1β, **(C)** IL-6, and **(D)** INF-*γ* in tissues were determined by real-time qPCR assay. *^##^p* < 0.01 vs. Normal group; *p < 0.05 and **p < 0.01 vs. DSS group. Homo, homoplantaginin.

It is well known that pro-inflammatory factors such as TNF-*α*, IL-1β, IL-6, and IFN-*γ* play important roles in colitis progression (15–17). The effects of Homo on the expression of pro-inflammatory factors were investigated using RT-qPCR technology. The expression levels of IL-6, IL-1β, TNF-α and IFN-γ mRNA in the colon tissue of the model group mice were significantly increased in comparison to the normal group. Treatment with (50 mg/kg) and 5-ASA (200 mg/kg) significantly inhibited the expression of TNF-α, IL-1β, IL-6, and IFN-γ mRNA (Figures 2A–D). It is worth noting that the effect of Homo (50 mg/kg) on IL-6, TNF-α and IL-1β was slightly better than that of 5-ASA (200 mg/kg). These results suggest that the inhibition of the occurrence and development of UC by Homo is closely related to its inhibitory effect on pro-inflammatory factors.

### 3.3 Homo inhibits the expression of chemokines and adhesion molecules in the colon of colitis mice

**Figure 3:**
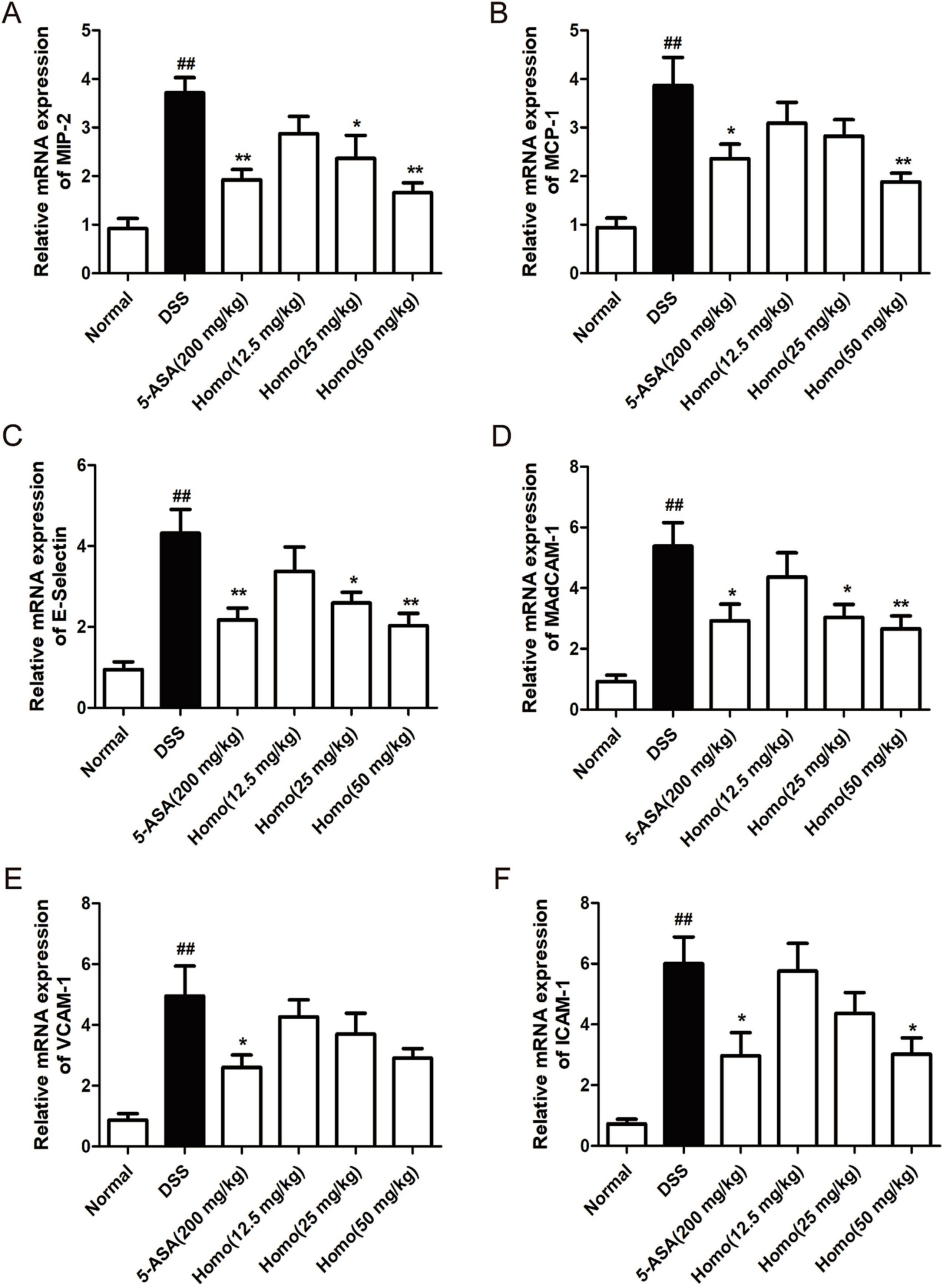
Effects of Homo on the expressions of chemokines and adhesion molecules in colonic tissues of mice with colitis. The mRNA expressions of chemokines **(A)** MIP-2, **(B)** MCP-1, adhesion molecules **(C)** E-selectin, **(D)** MAdCAM-1, **(E)** VCAM-1 and **(F)** ICAM-1were assessed by RT-qPCR (*n* = 6). *^##^p* < 0.01 vs. Normal group; *p < 0.05 and **p < 0.01 vs. DSS group. Homo, homoplantaginin.

Previous studies have demonstrated that chemokines and adhesion molecules play crucial roles in the progression of intestinal inflammation by regulating immune cell infiltration and activation, thereby contributing to the formation and development of inflammatory responses. In DSS-induced colitis, the continuous production of pro-inflammatory factors leads to a significant expression of chemokines and adhesion molecules. To evaluate the effects of Homo on the expression of these mediators, RT-qPCR was performed to detect the mRNA expression of MIP-2, MCP-1, E-selectin, MAdCAM-1, VCAM-1, and ICAM-1, and genes in colon tissues. As illustrated in Figures 3A–F, the model group mice exhibited significantly increased mRNA expression levels of chemokines (MIP-2 and MCP-1) and adhesion molecules (E-selectin, MAdCAM-1, VCAM-1, and ICAM-1) in their colon tissues in comparison to the normal group. Homo (50 mg/kg) significantly downregulated the mRNA expression of these genes. Similarly, 5-ASA (200 mg/kg) significantly suppressed their mRNA expression, demonstrating an effect comparable to that of Homo (50 mg/kg).

### 3.4 Homo protects the intestinal mucosal barrier in colitis mice

**Figure 4:**
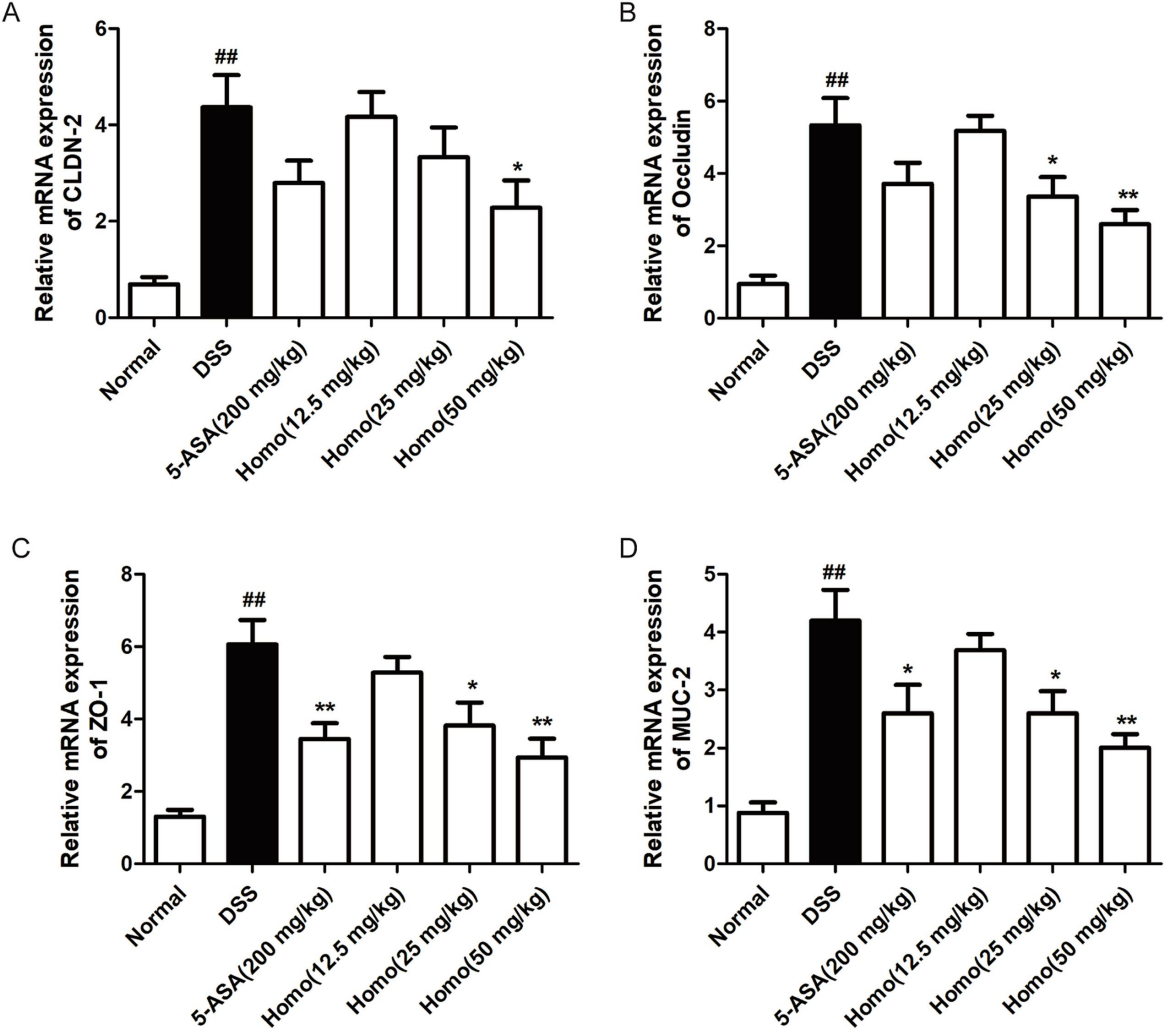
Effects of Homo on intestinal mucosal barrier integrity in colitis mice. The mRNA expressions of tight junctions **(A)** CLDN-2, **(B)** Occludin, **(C)** ZO-1, intestinal mucin **(D)** MUC-2 were assessed by RT-qPCR (*n* = 6). *^##^p* < 0.01 vs. Normal group; *p < 0.05 and **p < 0.01 vs. DSS group. Homo, homoplantaginin.

Extensive research has demonstrated that the integrity of the intestinal mucosal barrier is compromised, which subsequently alters the expression and function of chemokines and adhesion molecules, thereby exacerbating inflammation. To investigate the effects of Homo on the mucosal barrier in colon tissues, RT-qPCR was performed to assess the mRNA expression levels of CLDN-2, occludin, ZO-1, and MUC2 genes in the colon. As illustrated in Figures 4A–D, compared to the normal group, the colitis mice exhibited significantly increased mRNA expression levels of epithelial barrier proteins (CLDN-2, occludin, and ZO-1) as well as the mucin barrier protein (MUC2) in their colon tissues. Homo (50 mg/kg) significantly suppressed the mRNA expression of these genes. Notably, its effect was significantly superior to that of 5-ASA (200 mg/kg).

### 3.5 Homo down-regulates MMP9 mRNA level in colon tissue by targeting MMP9

**Figure 5:**
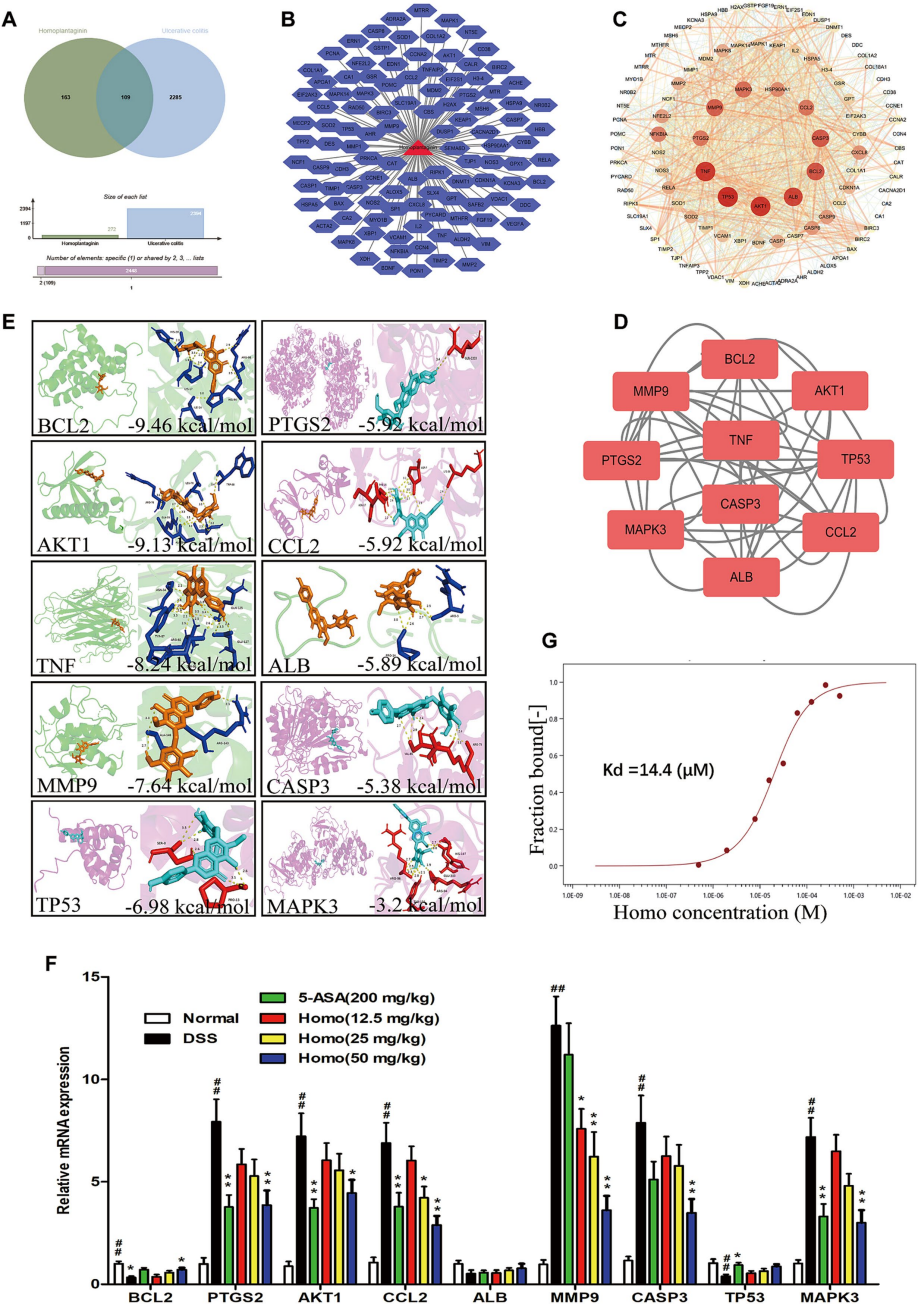
Homo inhibited colitis in mice by targeting MMP9. **(A)** Venn diagram of Homo and UC target. **(B)** Homo and 109 targets (Homo targets and UC targets intersection). **(C)** PPI network of Homo and UC targets. **(D)** The core target prediction of Homo intervention in UC. **(E)** Molecular docking visualization of Homo and the top 10 targets. **(F)** Effects of Homo on the mRNA expression of the top 10 targets in colon tissues of colitis mice. **(G)** The binding of Homo to MMP9 protein was detected by MST technology. (*n* = 6). *^##^p* < 0.01 vs. Normal group; *p < 0.05 and **p < 0.01 vs. DSS group. Homo, homoplantaginin.

To explore the target of Homo, bioinformatics was employed to analyze the target of Homo inhibiting the UC process. The results revealed 109 possible common targets between Homo and UC (Figures 5A,B). Among them, the top 10 core targets were identified as AKT1, TP53, TNF, ALB, BCL2, MMP9, CASP3, CCL2, PTGS2, and MAPK3 (Figures 5C,D). The results of virtual docking demonstrated that Homo exhibited high affinity for BCL2, AKT1, TNF, MMP9, and TP53 (Figure 5E). The above 10 core targets were detected by RT-qPCR. Moreover, the results indicated the significant regulatory effects of Homo on the expression of BCL2, PTGS2, AKT1, CCL2, MMP9, CASP3, and MAPK3. Notably, Homo inhibited the MMP9 mRNA expression in a dose-dependent manner, with a significant inhibitory effect observed at the dose of 12.5 mg/kg, suggesting that MMP9 may be primary target of Homo inhibiting the UC process (Figure 5F). To confirm this, MST analysis was performed to analyze the binding affinity of Homo to MMP9. The results demonstrated a strong binding interaction between Homo and MMP9 (Figure 5G). These findings indicate that Homo exerts its anti-colitis effect by targeting and inhibiting MMP9 expression.

### 3.6 MMP9/RLN2 signaling is involved in the process of Homo alleviating colitis in mice

**Figure 6:**
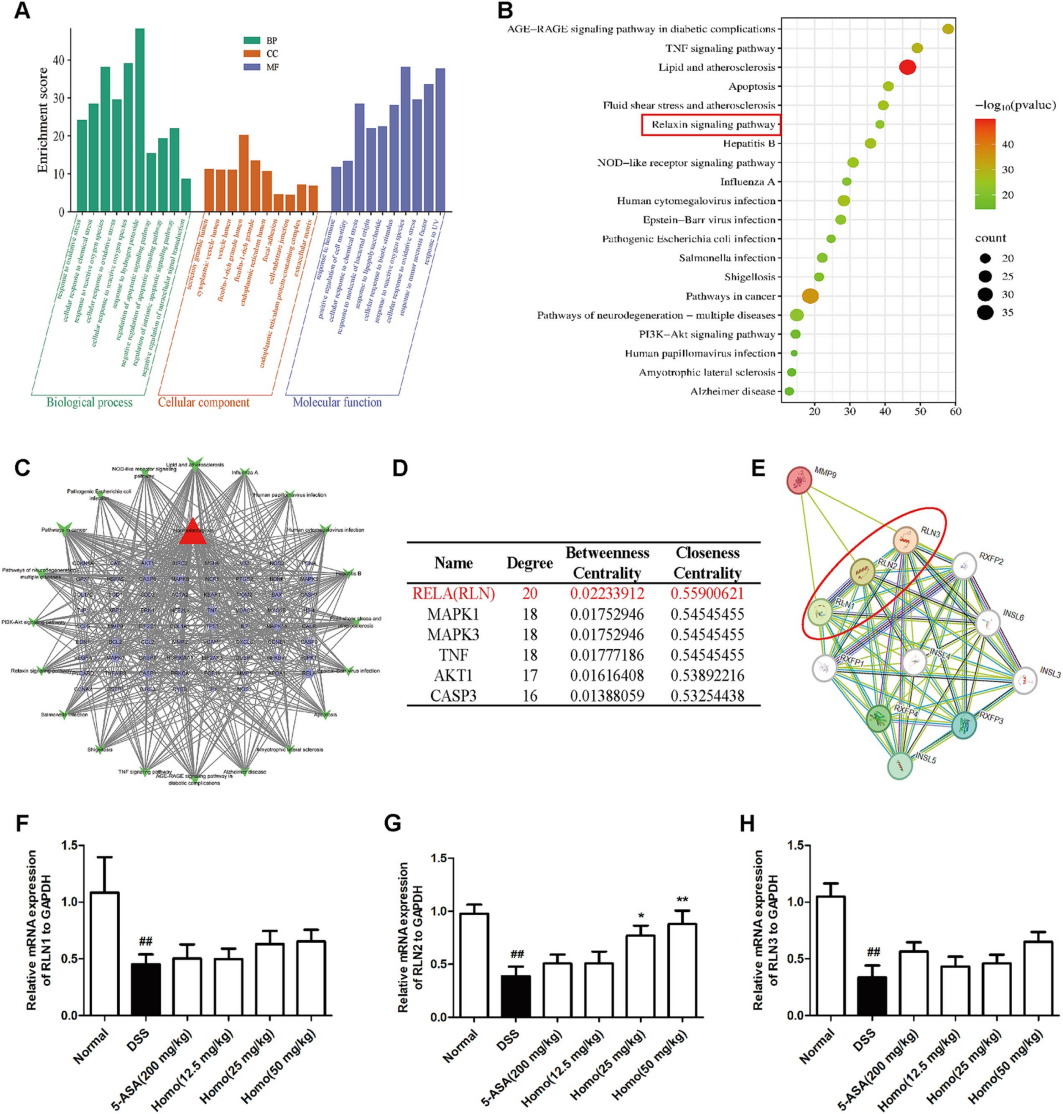
Effects of homo on MMP9-relaxin signaling. **(A)** GO and **(B)** KEGG pathway enrichment analysis. **(C)** Homo-targets-pathways network diagram. **(D)** The top 6 core pathways prediction of Homo intervention in UC. **(E)** The correlation between MMP9 and Relaxin signaling was predicted by STRING platform. **(F)**. The effect of Homo on the expression of **(F)** RLN1, **(G)** RLN2 and **(H)** RLN3 mRNA in colon tissues of colitis mice. *^##^p* < 0.01 vs. Normal group; *p < 0.05 and **p < 0.01 vs. DSS group. Homo, homoplantaginin.

To elucidate the signaling pathways underlying the effects of Homo on colitis, bioinformatics analysis was performed. The results indicated that the inhibitory effect of Homo on enteritis was closely associated with RLN, MAPK, TNF, AKT1, and CASP3 signaling pathways (Figures 6A–D). Among these pathways, the RELA signaling score was the highest. The correlation between MMP9 and RELA signaling pathways was analyzed using the STRING platform. The results revealed that MMP9 was highly correlated with the key signal molecules RLN1, RLN2, and RLN3 in the RELA pathway (Figure 6E). The effect of Homo on the mRNA expression of RLN1, RLN2, and RLN3 was examined using RT-qPCR. The results demonstrated that Homo upregulated RLN2 mRNA expression in a dose-dependent manner, while no significant effect was observed on RLN1 and RLN3 (Figures 6F–H). These findings suggest that Homo inhibits the development of colitis primarily by regulating the MMP9/RLN2 signaling pathway.

### 3.7 Homo improves the progression of colitis in mice by regulating the MMP9-RLN2 signaling axis

**Figure 7:**
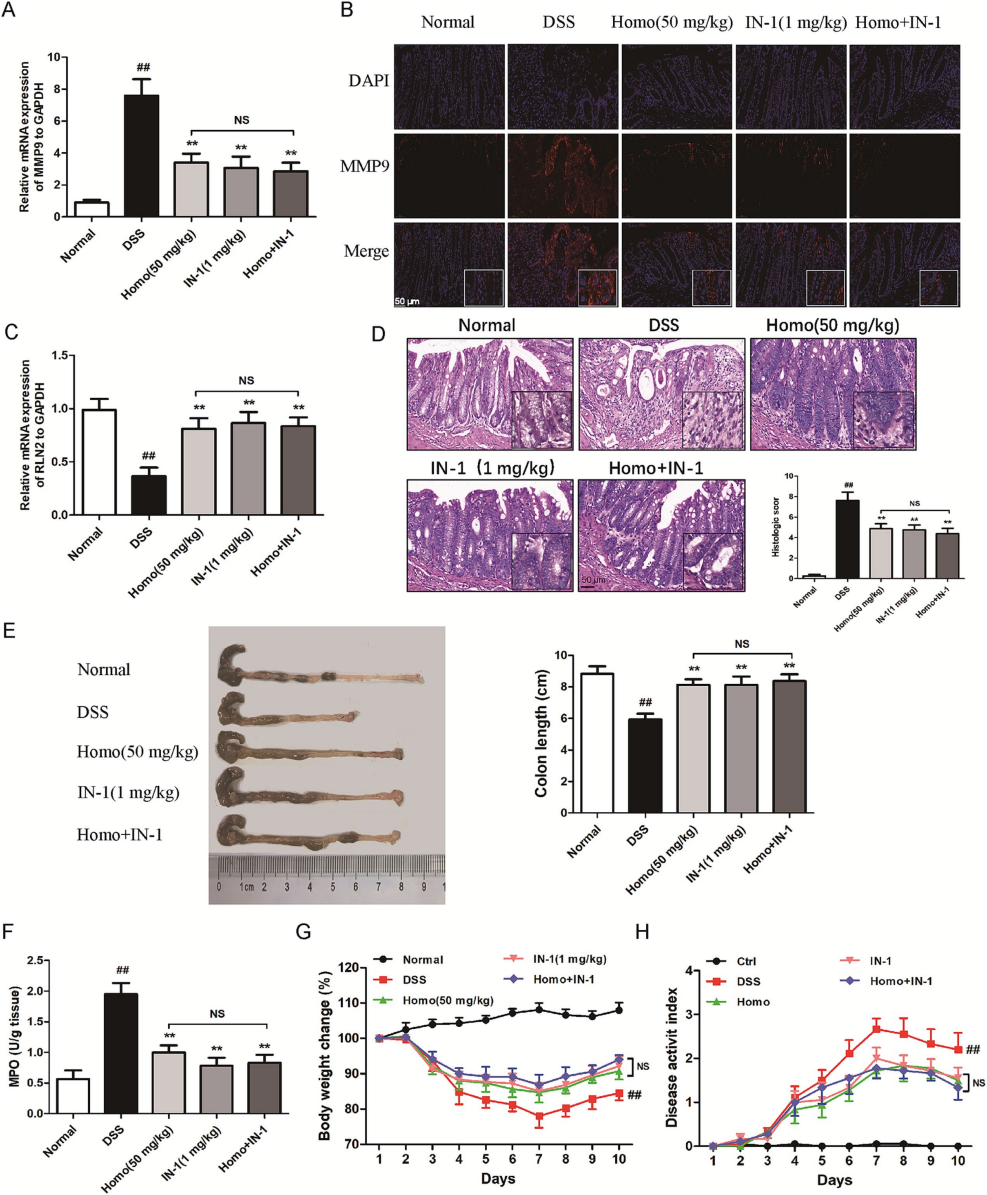
Homo inhibited the progression of colitis in mice by regulating the MMP9-RLN2 signaling axis. **(A)** The effect of Homo on the expression of MMP9 mRNA in colon tissues of colitis mice. **(B)** The protein expression of MMP9 was detected by tissue immunofluorescence staining (scale bar, 50 μm). **(C)** The effect of Homo on the expression of RLN2 mRNA in colon tissues of colitis mice. **(D)** H&E staining and histological score of colon tissues (scale bar, 50 μm). **(E)** The length of colons. **(F)** MPO activity in colon tissues. **(G)** Percent change of body weight. **(H)** The length of colons. (*n* = 6). *^##^p* < 0.01 vs. Normal group; *p < 0.05 and **p < 0.01 vs. DSS group. Homo, homoplantaginin.

To determine whether Homo inhibits the occurrence and development of colitis through the MMP9-RLN2 signaling pathway, the MMP9-specific inhibitor (IN-1, 1 mg/kg) in combination with Homo (50 mg/kg) was utilized to investigate its impact on colitis in mice. The results demonstrated that both IN-1 and Homo significantly suppressed MMP9 mRNA and protein expression (Figures 7A,B). Notably, the combination of IN-1 and Homo did not synergistically increase the inhibitory effect of Homo on MMP9. Both IN-1 and Homo significantly inhibited the RLN2 mRNA expression (Figure 7C). Additionally, both treatments significantly improved the pathological changes of colon tissue in mice, prevented colon shortening, reduced MPO activity, increased body weight, and lowered DAI score (Figures 7D–H). Interestingly, the combination of IN-1 and Homo did not synergistically increase the inhibitory effect of Homo on colitis in mice. In summary, these findings highlight that the MMP9-RLN2 signaling axis plays a crucial role in the inhibition of colitis by Homo.

## 4 Discussion

Inflammatory bowel disease, encompassing conditions such as UC and Crohn’s disease, represents a significant health challenge characterized by chronic inflammation of the gastrointestinal tract (18, 19). The etiology of IBD is multifactorial, involving a complex interplay of genetic predisposition, environmental factors, and dysregulated immune responses (20). The pathophysiological mechanisms of IBD involve alterations in the gut microbiome, immune dysregulation, and disruption of the intestinal barrier, ultimately leading to persistent inflammation and tissue damage (21). Despite advancements in treatment, including anti-inflammatory agents and biologics, achieving sustained remission remains a challenging, necessitating ongoing research into novel therapeutic strategies and targets that can effectively mitigate the disease procession and restore intestinal homeostasis (22).

The findings from this study demonstrate that Homo exhibits significant therapeutic effects on DSS-induced colitis in mice, primarily through its modulation of inflammatory pathways and the intestinal mucosal barrier. The observed reduction in body weight loss, reduced MPO activity, and improved colon morphology underscores the potential of Homo as a viable therapeutic agent for UC. The molecular mechanisms elucidated in this study suggest a multi-faceted approach, wherein Homo suppresses the expression of pro-inflammatory cytokines including, TNF-*α*, IL-1β, and IL-6, which are critical mediators in the pathogenesis of colitis. The inhibition of these cytokines not only alleviates the inflammatory response but also facilitates the restoration of epithelial integrity, thereby strengthening the mucosal barrier function.

The involvement of chemokines and adhesion molecules in the inflammatory cascade underscores the complexity of immune cell recruitment and activation in colitis (23). The significant downregulation of chemokines like MIP-2 and MCP-1, along with adhesion molecules including ICAM-1 and VCAM-1, indicates that Homo effectively modulates the inflammatory milieu, which is crucial for limiting immune cell infiltration into the colonic tissue. Furthermore, the findings suggest that Homo’s protective effects on the intestinal epithelial barrier are mediated through the modulation of tight junction proteins and mucins, which are essential for maintaining barrier integrity and function. The upregulation of ZO-1, Occludin, and MUC2 in the presence of Homo indicates its role in strengthening mucosal defense mechanisms (24). This restoration of barrier function is particularly significant, as a compromised intestinal barrier is a hallmark of IBD, contributing to dysregulated immune responses and exacerbated inflammation.

MMP-9 plays a critical role in the progression of UC (7, 25). Studies have demonstrated that MMP-9 expression is significantly elevated in patients with colitis, which is related to disease severity, suggesting its pro-inflammatory role in the inflammatory response (26). The role of MMP-9 in modulating pro-inflammatory cytokines such as TNF-*α*, IL-1β, and IL-6 further elucidated its pathogenic mechanism. MMP-9 not only activates the precursors of these factors, but also aggravates both local and systemic inflammatory responses by promoting immune cells infiltration. In addition, the effect of MMP-9 on the intestinal barrier is also critical (5, 7). It destroys the intestinal barrier and increases intestinal permeability by degrading the extracellular matrix, inhibiting the expression of connexins ZO-1 and occludin. Simultaneously, MMP-9 suppresses the production of mucin MUC2, thereby weakening the protective function of the intestine. In this study, we found that Homo selectively binds to MMP9 (27, 28). These findings indicate that Homo exerts anti-colitis effects by targeting and inhibiting MMP9 expression.

RLN2, a member of the Relaxin family, exhibits a wide range of anti-inflammatory effects, including roles in cardiovascular inflammation, airway, inflammation (29–32). Studies have demonstrated that RLN2 expression is downregulated in colitis models and negatively correlates with the severity of intestinal inflammation. This suggests that RLN2 may inhibit the inflammatory response, and play an important role in UC progression (33). Our findings demonstrate that Homo significantly up-regulates RLN2, with its mechanism closely related to MMP9 downregulation. This study highlights that targeting the MMP9/RLN2 signaling axis presents a promising approach to developing new strategies for UC treatment and other related gastrointestinal diseases. This study not only enhances our understanding of the potential mechanisms of colitis but also paves the way for the potential clinical application of Homo in UC treatment.

One of the limitations of this study is its dependence on a single animal model, especially the DSS-induced colitis mouse model, which may not fully summarize the complexity and heterogeneity of human UC. Although the therapeutic effects of Homo were promising, the results may not be directly translatable to clinical applications without further validation in other models or through clinical trials. Additionally, the mechanisms by which Homo modulates the MMP9-RLN2 signaling axis require extensive investigation. This includes exploring potential off-target effects and identifying other signaling pathways that may contribute to the observed therapeutic benefits. Future studies should also consider the long-term effects of Homo treatment and its safety profile to ensure its viability as a therapeutic option.

## 5 Conclusion

In summary, our findings provide strong evidence that Homo significantly ameliorates DSS-induced colitis in mice by regulating pro-inflammatory factors, chemokines, and adhesion molecules while preserving the intestinal mucosal barrier. The identification of MMP9 as a primary target, along with the involvement of the MMP9-RLN2 signaling axis, highlights the potential of Homo as a novel therapeutic agent for UC treatment. However, further research is required to elucidate the detailed mechanisms of action and to validate its efficacy and safety in clinical applications, paving the way for future therapeutic advancements in IBD management.

## Data Availability

The original contributions presented in the study are included in the article/supplementary material, further inquiries can be directed to the corresponding authors.

## Footnotes

^1^ https://pubchem.ncbi.nlm.nih.gov/

^2^ https://ctdbase.org/

^3^ http://swisstargetprediction.ch/

^4^ https://www.genecards.org/

^5^ https://omim.org/

^6^ http://www.bioinformatics.com.cn/?p=1

^7^ https://cn.string-db.org/

^8^ https://david.ncifcrf.gov/
